# Clinical Pharmacogenetics of Cytochrome P450-Associated Drugs in Children

**DOI:** 10.3390/jpm7040014

**Published:** 2017-11-02

**Authors:** Ida Aka, Christiana J. Bernal, Robert Carroll, Angela Maxwell-Horn, Kazeem A. Oshikoya, Sara L. Van Driest

**Affiliations:** 1 Department of Pediatrics, Vanderbilt University Medical Center, Nashville, TN 37232, USA; ida.t.aka@vanderbilt.edu (I.A.); christiana.j.bernal@vanderbilt.edu (C.J.B.); angela.c.maxwell-horn@vanderbilt.edu (A.M.-H.); kazeemoshikoya@ymail.com (K.A.O.); 2 Department of Biomedical Informatics, Vanderbilt University Medical Center, Nashville, TN 37232, USA; robert.carroll@vanderbilt.edu; 3 Department of Medicine, Vanderbilt University Medical Center, Nashville, TN 37232, USA

**Keywords:** pediatrics, pharmacogenomics, electronic health records, drug–gene interactions, cytochrome P450

## Abstract

Cytochrome P450 (CYP) enzymes are commonly involved in drug metabolism, and genetic variation in the genes encoding CYPs are associated with variable drug response. While genotype-guided therapy has been clinically implemented in adults, these associations are less well established for pediatric patients. In order to understand the frequency of pediatric exposures to drugs with known CYP interactions, we compiled all actionable drug–CYP interactions with a high level of evidence using Clinical Pharmacogenomic Implementation Consortium (CPIC) data and surveyed 10 years of electronic health records (EHR) data for the number of children exposed to CYP-associated drugs. Subsequently, we performed a focused literature review for drugs commonly used in pediatrics, defined as more than 5000 pediatric patients exposed in the decade-long EHR cohort. There were 48 drug–CYP interactions with a high level of evidence in the CPIC database. Of those, only 10 drugs were commonly used in children (ondansetron, oxycodone, codeine, omeprazole, lansoprazole, sertraline, amitriptyline, citalopram, escitalopram, and risperidone). For these drugs, reports of the drug–CYP interaction in cohorts including children were sparse. There are adequate data for implementation of genotype-guided therapy for children for three of the 10 commonly used drugs (codeine, omeprazole and lansoprazole). For the majority of commonly used drugs with known CYP interactions, more data are required to support pharmacogenomic implementation in children.

## 1. Introduction

The cytochrome P450 (CYP) families of enzymes are major players for phase I metabolism of drugs. It is estimated that the CYP3A family metabolizes approximately one-third of all drugs, CYP2D6 about one-fourth of all drugs, and other CYPs over one-tenth of all drugs [1]. For many of these enzymes, there are both common and rare variants in the genes encoding the enzymes that lead to functional alterations, causing gain of function or loss of function phenotypes. These naturally occurring variants lead to differences in drug and metabolite concentrations. In instances where the therapeutic window is narrow and a critical step in the drug metabolism pathway involves a polymorphic gene, there is a potential for a drug–gene interaction (DGI). In other words, genotype may explain individual variability in response. In these instances, pre-prescription testing to determine the genotype may be a viable strategy for selecting the right drug and dose for individual patients, with the hopes of maximizing therapeutic benefit while minimizing risk of untoward effects [2,3,4,5,6,7,8,9,10,11].

Given the motivation for use of genomic information to guide prescribing and the large number of studies to date exploring potential DGIs, the Clinical Pharmacogenomic Implementation Consortium (CPIC) and Pharmacogenomics Knowledge Base (PharmGKB) resources have developed schemas for critical evaluation of evidence for drug–gene associations and clinical utility of genotype information for prescribers [12,13]. PharmGKB levels of evidence range from 1 (highest) to 4 (lowest), and are assigned based on robustness of the data in support of each specific DGI. For example, DGIs are assigned to level 1 if associations are replicated in multiple distinct cohorts with statistically significant *p*-values, while level 4 evidence comes from case reports or in vitro data. CPIC levels add a focus on clinical actionability and prioritization for implementation. For example, level A DGIs have a high level of supporting evidence and there is a recommendation to change prescribing based on genetic information. Given their key role in drug metabolism, CYP enzymes represent a large portion of the DGIs cataloged in these resources; genes coding for CYPs are involved in 1241 of the 7395 DGIs on PharmGKB, and 104 of the 352 on the CPIC website [14,15].

Absent from either the PharmGKB or CPIC evaluation criteria are considerations for specific patient populations. In particular, for pediatric patients, the indications for use of individual drugs are often different than in adults, side effect profiles vary by age, and ontogeny of the CYP enzymes, particularly for infants, can impact the drug–gene relationship [16]. Furthermore, compared to the body of evidence from studies in adults, there is a paucity of research supporting the use of genotype to guide prescribing in children.

Due to the potential for clinical pharmacogenomic testing and the lack of pediatric specific data, the goals of this manuscript are as follows: first, to use a single-center, longitudinal, electronic health record (EHR) cohort to determine the frequency of pediatric exposures to medications with established interactions with CYP enzymes (i.e., define the number of unique pediatric patients exposed to drugs with CPIC level A or B and PharmGKB level 1 or 2 evidence for the drug–CYP association); to review the current pediatric literature for CYP-associated drugs most frequently used in pediatric patients; and to make recommendations regarding implementation of CYP pharmacogenomics for pediatric patients.

## 2. Results

### 2.1. CYP-Associated Drugs with High Levels of Evidence

**Figure 1:**
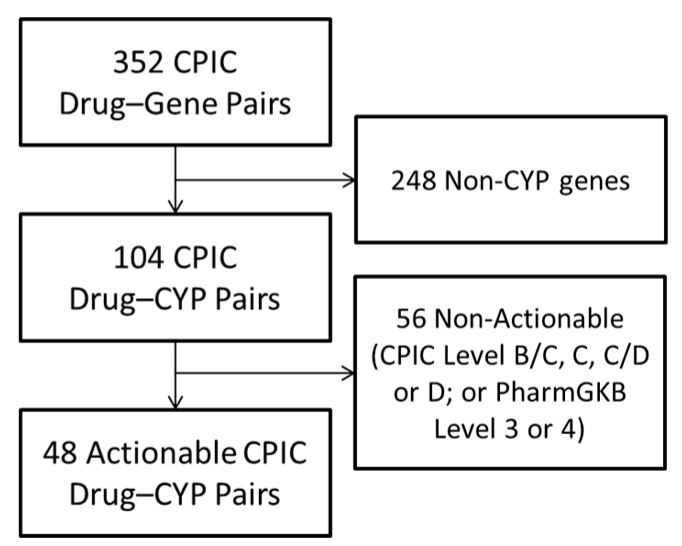
Selection of Actionable Drug–Gene Pairs. Of the 352 Drug–gene pairs categorized on the Clinical Pharmacogenomics Implementation Consortium (CPIC) website, 104 represented genes encoding Cytochrome P450 (CYP) enzymes, and 48 (representing 41 unique drugs) were deemed actionable, defined as having CPIC Level A or B and PharmGKB level 1 or 2 evidence. DGI—Drug–gene interaction; PharmGKB—Pharmacogenomics Knowledgebase.

The CPIC drug–gene pairs table included a total of 352 DGIs, representing 223 unique drugs and 127 unique genes. Filtering for CYP genes and a high level of CPIC and PharmGKB evidence resulted in 48 actionable drug-CYP pairs, including 41 unique drug names (Figure 1).

### 2.2. Pediatric Exposures to 41 CYP-Associated Drugs

Using the Synthetic Derivative (SD), the Vanderbilt University Medical Center de-identified database of EHR data, we performed a search for the number of unique individuals exposed to each of the 41 CYP-associated drugs when age <18 years and identified subsets of CYP-associated drugs that are used commonly, moderately and rarely in pediatric patients (Table 1). Over 5000 children were exposed to the drugs over the decade-long time period of the query for 10 of the CYP-associated drugs (ondansetron, oxycodone, codeine, omeprazole, lansoprazole, sertraline, amitriptyline, citalopram, risperidone and escitalopram). The age distribution at the time of first exposure varied by drug, with some drugs (e.g., methadone) predominately used in infants, others in early childhood (e.g., ondansetron), and others in adolescence (e.g., sertraline). The race and ethnicity of the exposed patients, obtained from administrative data, approximated that of the patient population as a whole, except for drugs with specific indications that are disproportionately present by race. For example, the antiviral drugs nevirapine and efavirenz, used to treat HIV, had higher percentages of Black/African American patients, consistent with higher rates of HIV infection seen in these children, although the majority of children exposed to these drugs were of white or unknown race [17]. 

### 2.3. Review of the Literature Supporting Drug–CYP Interactions for Drugs Commonly Used in Children

We performed a review of the literature, searching for primary data sources with pediatric research participants for each of the 10 CYP-associated drugs most frequently used in children. A summary of the literature reviewed is provided in Table 2. In all, 38 unique relevant manuscripts that involved pediatric participants were included; two manuscripts described multiple DGIs, and data relevant for each are included in the table. The number of manuscripts supporting each CYP–drug interaction varied from 0 (for ondansetron and sertraline) to 15 (for risperidone). The number of individuals comprising the cohort for each study also varied, from case reports of one to three individuals (six such reports for codeine and one for amitriptyline) to cohorts of 19 to 830 children. The median number of study participants across all studies was 72 individuals (interquartile range (20–120) individuals), and for cohort studies was 84 (38–133) individuals. Despite the small cohort size, the majority (20/30) of cohort studies reported at least one significant association of a CYP to a pharmacokinetic or pharmacodynamic outcome. 

#### 2.3.1. Ondansetron

Ondansetron is a 5-hydroxytryptamine type 3 (5-HT3) receptor antagonist used in adults and children to prevent nausea and vomiting in the setting of chemotherapy, radiation and surgery. The mechanism of action includes binding of the drug to central and peripheral 5-HT3 receptors to prevent serotonin-meditated emetogenic signaling. Intravenous ondansetron is approved for use in patients six months of age and older for nausea and vomiting due to emetogenic cancer chemotherapy and for patients aged one month and older for postoperative nausea and vomiting [56]. The oral ondansetron drug label includes dosing information for ages ≥4 years [57]. Ondansetron is also used off-label in the treatment of nausea and vomiting in a variety of settings including gastroenteritis in patients over one month of age.

The metabolism of ondansetron involves CYP3A4, CYP1A2 and CYP2D6, as well as glucuronide conjugation to inactive metabolites. Multiple studies have linked variability in CYP2D6 function to the pharmacokinetics of ondansetron and drug response in adults (reviewed in [58]); CYP2D6 ultra-rapid metabolizers have lower ondansetron exposure and less efficacy. Therefore, the CPIC guideline recommends alternate antiemetics that are not CYP2D6 substrates (e.g., granisetron) for individuals who are known CYP2D6 ultra-rapid metabolizers. Of note, tropisetron is not a good choice as an alternative agent, as it is also predominately metabolized by CYP2D6. There are no data from studies including pediatric patient populations to support or refute the ondansetron–CYP interaction in children. CYP2D6 function increases rapidly in the first month after birth, thus it is expected that *CYP2D6* genetic variation will similarly affect ondansetron response in children as adults, alternative age-specific metabolic pathways notwithstanding [58,59].

#### 2.3.2. Oxycodone and Codeine

Oxycodone and codeine are opioid analgesics used to relieve pain when alternative non-opioid treatment options are inadequate. Codeine is a naturally occurring methylated morphine compound that is metabolized by CYP2D6 to morphine, which then binds to μ-opioid receptors to effect pain relief. Oxycodone is a semisynthetic opioid, and although the parent compound can bind to μ-opioid receptors, the O-demethylated CYP2D6 metabolite of oxycodone binds with much higher affinity and potency. Single-ingredient codeine and all oxycodone-containing products are approved by the US Food and Drug Administration (FDA) only for use in adults [60,61]. Codeine with acetaminophen was approved by the FDA in patients over three years of age [62]. However, the FDA announced in April 2017 that due to safety concerns, the drug label for all codeine-containing products must include a Contraindication (the FDA’s strongest warning), stating that codeine should not be used to treat pain or cough in children younger than 12 years, a new Warning that codeine should not be used in adolescents 12 to 18 years of age who are obese or have obstructive sleep apnea or severe lung disease, and a strengthened Warning to mothers that breastfeeding is not recommended when taking codeine. The codeine label also includes black box warnings stating that respiratory depression and death have occurred in children who received codeine following tonsillectomy and/or adenoidectomy, and that CYP2D6 inhibitors may impact drug response. Both codeine and oxycodone have been widely used in pediatric patients for analgesia.

As a prodrug that requires CYP2D6 metabolism to the active compound, codeine is a prototype for drug–CYP interaction. Individuals who lack CYP2D6 function (poor metabolizers) are unable to convert codeine to morphine, and thus have no therapeutic effect. Conversely, CYP2D6 ultra-rapid metabolizers, who have more than two functional copies of the *CYP2D6* gene, are able to generate excess morphine and are at risk for toxicity, including respiratory depression and death. Although much of high-quality evidence for the CYP–codeine interaction comes from adults (reviewed in [63,64]) there is ample evidence from studies including pediatric patients to demonstrate the clinical impact of *CYP2D6* genotype in children. There are several case reports of infant mortality and respiratory failure after exposure to codeine either through breast milk or for analgesia after tonsillectomy/adenoidectomy. In many of these cases, the infants or children (and/or their mother in the cases of exposure through breast milk) were found to be CYP2D6 ultra-rapid metabolizers [21,22,24,25,26,29]. Furthermore, cohort and case-control studies exploring the pharmacokinetics and pharmacodynamics of codeine in children have confirmed higher morphine levels and risk for adverse events in children with ultra-rapid CYP2D6 function [19,20,23,27,28,30]. There is one report of clinical implementation of *CYP2D6* genotyping for pediatric patients with sickle cell disease in order to provide genotype-guided therapy; clinical genetic testing coupled with decision support resulted in no patients with ultra-rapid or poor metabolizer genotypes being prescribed codeine [31]. There are strong data supporting the impact of CYP2D6 on codeine response in children. The FDA contraindication for use in children precludes any use of codeine in patients under 12 years of age and some adolescents. For patients who are ≥12 years of age, if codeine therapy is considered, CYP2D6 metabolizer status should be determined in order to prevent inefficacy and toxicity.

The evidence for the impact of *CYP2D6* variation on oxycodone response is less robust. Although differences in the pharmacokinetics of oxycodone based on CYP2D6 metabolizer status are evident, there are contradictory data regarding the differences in analgesia or toxicity (reviewed in [63,64]). There is one report of the impact of CYP2D6 metabolizer status on oxycodone pharmacokinetics in adolescents, where results were consistent with adult pharmacokinetic studies [18]. Although this study confirmed that CYP2D6 normal metabolizers had higher oxymorphone exposure than poor or intermediate metabolizers, more data are needed to support the oxycodone– CYP interaction before genotype-guided oxycodone dosing is clinically implemented for children. 

#### 2.3.3. Omeprazole and Lansoprazole

Proton pump inhibitors (PPIs) are one of the highest-selling prescription medication classes, both in the United States and globally, with growing popularity among pediatric practitioners [65]. The mechanism of action targets the gastric cells, covalently binding to and irreversibly inactivating the proton (acid) pump, thereby suppressing acid secretion. While the primary indication for PPI use has historically been gastroesophageal reflux disease (GERD), PPIs are routinely prescribed for a variety of upper intestinal tract conditions and are increasingly utilized in chronic respiratory diseases as well [66,67]. Despite their broad use, the only FDA-approved indications for PPI use in children are short-term treatment of symptomatic GERD and treatment of eosinophilic esophagitis [68,69,70,71,72,73]. Furthermore, of the five available PPI formulations approved for pediatric use, esomeprazole is the only drug approved by the FDA for use in children less than one year of age. Thus, the use of omeprazole and lansoprazole, the two most commonly prescribed PPIs for the treatment of GERD in infants, is off-label and outside of the treatment guidelines [74].

PPIs are metabolized primarily by the liver microsomal enzyme CYP2C19 and to a lesser extent CYP3A4. Genetic variations in the *CYP2C19* gene give rise to metabolizer phenotypes with varying degrees of PPI clearance. Individuals with loss of function alleles are termed poor metabolizers and experience reduced drug clearance relative to the normal metabolizer phenotype [75]. Conversely, ultra-rapid metabolizers have gain of function alleles which confer increased rates of clearance and reduced drug exposure compared to normal metabolizers [76]. These pharmacokinetic associations have been demonstrated repeatedly in the adult population. For example, drug exposure as represented by the area under the plasma concentration versus time curve (AUC) for omeprazole, lansoprazole, and pantoprazole is 4- to 15-fold higher in poor metabolizers than in normal metabolizers [77]. Additionally, poor metabolizers exposed to omeprazole have higher (less acidic) intra-gastric pH than normal and intermediate metabolizers, supporting the role *CYP2C19* genetic polymorphisms play in PPI efficacy [78]. 

Several pediatric studies have also validated the PPI and *CYP2C19* drug–gene interaction, with pantoprazole and lansoprazole being the two most investigated drugs. Children identified as poor metabolizers treated with therapeutic doses of pantoprazole or lansoprazole have significantly higher AUCs, delayed clearance, and longer drug half-life than normal metabolizers [32,33,79,80,81]. In addition to these pharmacokinetic parameters, clinical outcomes of efficacy and adverse events have also correlated with metabolizer phenotypes, particularly for lansoprazole [33,34,35]. While some data fail to support a gene–dose relationship for omeprazole [32], a recent study demonstrated increased acid exposure (decreased PPI efficacy) in ultra-rapid metabolizers as compared to normal, intermediate, and poor metabolizers, collectively [36]. While more studies are needed to further characterize the impact of *CYP2C19* polymorphisms on PPI therapy, there are sufficient data to support pharmacogenomic implementation in pediatric patients.

#### 2.3.4. Sertraline

Sertraline is an antidepressant in the Selective Serotonin Reuptake Inhibitor (SSRI) class. The mechanism of action for SSRIs is to prevent the reuptake of serotonin by presynaptic receptors, which in turn increases the amount of serotonin available to bind to the postsynaptic receptors. Sertraline is FDA-approved to treat major depressive disorder, obsessive–compulsive disorder, panic disorder, post-traumatic stress disorder, social anxiety disorder and premenstrual dysphoric disorder in adults and obsessive–compulsive disorder in children 6 years old and older [82]. The FDA label for SSRIs includes a black box warning due to an increased risk of suicidal thoughts and behavior in pediatric and young adult patients. Sertraline is commonly used off-label for childhood depression with close monitoring for suicidal thoughts.

Sertraline is metabolized to desmethylsertraline by several cytochrome P450 enzymes, including CYP2B6, CYP2C19, CYP2C9, CYP2D6 and CYP3A4. Sertraline is also a moderate inhibitor of CYP2D6. Decreased CYP2C19 function (due to concomitant inhibitor or poor metabolizer genotype), leads to increased plasma levels of the drug [83,84]. However, the results from studies of the impact of specific CYP gene variants to treatment outcomes are not consistent [85,86,87,88]. Despite this limited evidence, both CPIC and the Pharmacogenetics Working Group of the Royal Dutch Pharmacists recommend a 50% dose reduction of sertraline in CYP2C19 poor metabolizers [89]. There are no pediatric studies evaluating genetic variants or metabolizer phenotypes of CYP2B6, CYP2C19, CYP2C9, CYP2D6 and CYP3A4 with treatment outcomes. More research needs to be done in this area before routine pharmacogenetic testing can be recommended to optimize sertraline dosing in children.

#### 2.3.5. Amitriptyline

Amitriptyline is a tertiary amine tricyclic antidepressant (TCA). The mechanism of action of TCAs includes inhibition of reuptake of both serotonin and norepinephrine. Amitriptyline is FDA-approved for the treatment of depression in adults and adolescents over 12 years of age, with a black box warning due to the increase in suicidality for young patients [90]. Off-label uses in children include migraine prophylaxis, as an adjunctive therapy for chronic neuropathic pain, and for symptomatic management of pain-predominant functional gastrointestinal disorders.

Amitriptyline is metabolized by CYP2C19 to pharmacologically active secondary amines, which have more predominant noradrenergic effects than the parent compound. Both amitriptyline and the secondary amine metabolites are metabolized by CYP2D6 to inactive compounds. Variation in CYP2D6 function is hypothesized to impact drug clearance, while CYP2C19 variability contributes to individual differences in response by affecting the balance of noradrenergic and serotonergic effects. In adults being treated for depression, dose reduction or alternate therapy is recommended by CPIC if the patient is known to be a CYP2C19 or CYP2D6 poor metabolizer [91]. Alternate therapy is also recommended for CYP2C19 or CYP2D6 ultra-rapid metabolizers due to the risks for inefficacy or side effects. Our literature search revealed one case report of a 6-year-old female who survived chronic overdose of amitriptyline (10-fold typical dosing administered nightly for one month); genotyping in this individual revealed normal metabolizer status for both CYP2D6 and CYP2C19, which may have prevented a more disastrous outcome for the patient [37]. Whether or not the genotype-guided dosing recommendations for treating depression in adults are appropriate for other indications and/or pediatric patients has not been rigorously assessed. 

#### 2.3.6. Citalopram and Escitalopram

Citalopram and escitalopram are SSRIs widely used for the treatment of depression, anxiety disorders, and obsessive–compulsive disorder. Citalopram is a racemic mixture of *R*- and *S*-enantiomers, while escitalopram contains only the pharmacologically active *S*-enantiomer. Citalopram is approved for the treatment of depression in adults, and escitalopram is approved for the treatment of anxiety in adults and depression in patients over 12 years of age [92,93]. As with sertraline, both drug labels include a black box warning due to increased risk for suicidal thinking and behavior in young patients. Both drugs are frequently used off-label in children with depression, anxiety, obsessive–compulsive disorder, autism, and/or pervasive developmental disorders [38,89,94].

Both drugs are primarily metabolized by CYP2C19 and to a lesser extent by CYP2D6 and CYP3A4 [95]. CPIC guidelines recommend a 50% dose reduction or alternative drug not metabolized by CYP2C19 for poor metabolizers, as CYP2C19 poor metabolizers have higher plasma concentrations and this may increase the probability of side effects [89]. In pediatric patients, *CYP2C19* genotyping was conducted in a study investigating citalopram and metabolite concentrations at steady state in 19 adolescents, two-thirds of whom were >18 years of age; individuals with *CYP2D6* and/or *CYP2C19* variants were not significantly different from those without variants, although numbers were small (*n* = 3 CYP2D6*4 heterozygotes, *n* = 2 *CYP2D6* duplication, *n* = 3 CYP2C19*2 heterozygotes) [38]. An additional study of 83 adolescent and adult patients treated with citalopram or escitalopram demonstrated that CYP2C19*2 carriers had higher drug concentration to dose ratios, indicating impaired drug metabolism [39]. An investigation of the clinical endpoint of improvement in irritability among 89 pediatric and adult patients with autism spectrum disorder treated with escitalopram found no difference by CYP2C19 metabolizer status, although a secondary analysis demonstrated slower rate of change in dose over time for CYP2C19 ultra-rapid metabolizers [40]. Based on these mixed results from small studies, pharmacogenomic-guided dose titration for citalopram and escitalopram is not yet well supported in children.

#### 2.3.7. Risperidone

Risperidone, a serotonin-dopamine antagonist, is one of the atypical antipsychotic drugs used for treating schizophrenia, bipolar mania, autism and other impulsive or aggressive behaviors mostly in adults [96]. Risperidone, like other atypical antipsychotic drugs, has affinity for dopamine (D2), serotonin (5-HT2A), alpha adrenergic (α-1 and α-2), and histamine (H1) receptors. The mechanism of action of risperidone is not fully understood but current theories focus mainly on its inhibitory effects on D2 and 5-HT2A receptors [97,98]. The drug is metabolized in the liver primarily by CYP2D6 and to a lesser extent by CYP3A4 via hydroxylation to 9-hydroxyrisperidone, an equipotent metabolite [96,99]. *CYP2D6* variation is known to impact this metabolic transformation [100]. Risperidone is FDA-approved for use in children 5–16 years of age to treat irritability associated with autism, children 10–17 years to treat mania and mixed state due to bipolar disorder, and children 13–17 years to treat schizophrenia [101]. The drug is also increasingly used off-label for conditions including developmental and disruptive disorders, depression, obsessive–compulsive disorder, post-traumatic stress disorder, personality disorder, attention deficit-hyperactivity disorder, and Tourette’s syndrome [102]. 

As a major substrate of CYP2D6, risperidone has a potential for drug–CYP interaction. *CYP2D6* variants may contribute to an increased risk of adverse events associated with risperidone therapy. CYP2D6 poor metabolizers have prolonged drug exposure and therapeutic effect and may be at risk for toxicity [103]. In contrast, CYP2D6 ultra-rapid metabolizers, who have duplicate or multiple functional copies of the *CYP2D6* gene, may be at risk for therapeutic failure. There are few studies in adults supporting CYP–risperidone interaction with respect to pharmacokinetics, efficacy, and side effects [104,105,106,107,108,109], whereas others have failed to find risperidone–CYP associations [110]. Some studies have also demonstrated the clinical impact of *CYP2D6* genotype in children. In a small study involving children with autism, those who were CYP2D6 poor metabolizers had higher drug concentration and exhibited adverse effects including hyperprolactinemia and tardive dyskinesia, while ultra-rapid metabolizers exhibited no adverse effect [47]; however, the differences in drug response were not statistically significant. A cohort study evaluating the pharmacokinetics and pharmacodynamics of risperidone in children with pervasive developmental disorder did not demonstrate any clinically significant adverse effect of hyperprolactinemia after 8 weeks of risperidone therapy [41]. In the same study, serum prolactin level after 8 weeks of therapy was positively correlated with risperidone dosage, number of functional *CYP2D6* genes, and serum 9-hydroxyrisperidone, but not with risperidone plasma levels. In another study, children who were CYP2D6 ultra-rapid metabolizers exhibited less weight gain while on risperidone therapy compared to normal metabolizers; however, poor metabolizers showed similar effect to the normal metabolizers [43]. Other studies evaluating the effects of major and minor CYP2D6 inhibitors, rather than *CYP2D6* genotype, on the metabolism of risperidone in children demonstrated a much stronger association of CYP2D6 function to plasma levels of risperidone than with its major metabolite [111]. The Dutch Pharmacogenetics Working Group has recently changed its dosing recommendations of risperidone to “no action is required” for CYP2D6 poor metabolizers. The evidence for the impact of *CYP2D6* variability on the efficacy and adverse effects of risperidone is insufficient to support changes in prescribing based on genotype at this time. 

## 3. Discussion

Beginning with the compendium of drug–gene interactions from CPIC, we identified 48 actionable drug–CYP pairs, representing 41 distinct drugs, with high-quality evidence. Of those, the majority of drugs are rarely used in pediatric patients; only 10 of the 41 drugs had over 5000 individual pediatric patients exposed over a 10-year period at this tertiary children’s medical center. For these 10 drugs with a high level of evidence for clinical use of pharmacogenomic information in adults and over 500 pediatric patients exposed per year, we surveyed the literature to determine what data support implementation of genome-guided prescribing in children. Despite relatively high use of these medications in children, few drugs have robust evidence for the drug–CYP interaction in children: codeine (for which the FDA has now issued a contraindication against use in children under 12 years of age) and the PPIs, including omeprazole, lansoprazole and pantoprazole. For the remaining drugs, there are little data or conflicting data for pediatric patients.

Pharmacogenomic associations discovered in adults may be applied to pediatric patients in some circumstances. Specifically, when the gene is expected to have the same functional impact in children as adults and the indication and side effect profile for the drug are the same for children as adults, guidelines such as CPIC can be used in pediatrics, as suggested for ondansetron. It is important to pay heed to how children and adolescents differ from adults [112]. There are several issues that may preclude the extrapolation of drug–gene interaction from adults to children. One important issue is the effect of gene ontogeny, or the developmental regulation of gene expression. Many CYP enzymes are not expressed, or expressed at very low levels, in the neonatal period, with increases to the equivalent of adult levels of expression over weeks to years [113]. However, expression of *CYP2D6* and *CYP2C19*, the genes with the most potential clinical relevance at this time, reach levels equivalent to adults during infancy and then are stable through childhood and adolescence. Thus, the issue of ontogeny is most important for drugs given in the neonatal period, such as PPIs. Less is known about the unique CYP enzymes that are up-regulated in infancy, childhood and adolescence. In addition to the issue of ontogeny, many drugs are used in children for different indications than in adults (e.g., non-steroidal anti-inflammatory drugs to close patent ductus arteriosus in neonates). The drug–CYP interactions defined from adult studies may or may not be relevant to the clinical use of the drug for these pediatric indications. Pediatric patients may also have a unique side effect profile (e.g., adolescents and young adults are at increased risk for suicidal ideation with antidepressant therapy). These age-specific side effects may also have age-specific drug–gene interactions that modify the risk of their occurrence. For all these reasons, it is important to study pharmacogenomic associations in children prior to clinical implementation, unless there are robust data to indicate that ontogeny is not an issue, the indication for drug use is the same in adults and children, and the side effect profile is the same in adults and children. Indeed, the same logic can be applied across special populations, whether they are defined by age, ancestry, comorbid conditions, or any other stratifying factor.

Several themes are apparent from our focused literature review. First is that for each of the drug–CYP associations, there are a small number of studies available that include pediatric participants, despite the fact that we focused on the most commonly used medications. Second, each study generally included a small number of participants. This is particularly problematic for studies reporting negative findings, as the study may not have sufficient power to detect a clinically meaningful difference between groups, especially if the genotype of interest is infrequently found in the population. Third, a wide variety of primary outcomes were used, from drug levels to biomarkers to measures of drug efficacy or adverse events. This may also contribute to apparently contradictory results across studies. There was also a wide variety with respect to interrogation of the gene of interest. For example, some studies genotyped selected SNPs in *CYP2D6* and searched for association, while others genotyped many SNPs across the entire gene, as well as assessing for deletion and duplication. While it may be appropriate to focus genotyping efforts on the most common variants in the specific population being studied, false negative results may also stem from incomplete interrogation of the gene of interest. Thus, in addition to a paucity of studies in pediatric patients, there is a lack of high-quality, rigorous studies from which to draw conclusions.

Our approach to this topic has several limitations. We began our analysis with the compendium of CPIC drug–gene interactions, which is not a complete assessment of all drugs and all pharmacogenomic observations. There are several drug–gene interactions that are well-established and relevant in the care of pediatric patients, such as thiopurine drugs and *TPMT*, that are not included in CYP-focused review [114,115]. There are also several CYP-associated drugs with pediatric evidence that fell below our threshold for focused literature review, including atomoxetine, which has pediatric evidence for *CYP2D6-*guided therapy, and warfarin, for which there are pediatric-specific dosing calculators using *CYP2C9* and *VKORC1* genotypes [116,117]. We also used data from a single tertiary referral children’s hospital (which includes both inpatient orders and outpatient prescriptions) in order to assess for rates of exposure in a 10-year period. These data are subject to local practice patterns and regional trends. Our use of data from the last 10 years may also affect results, as new drugs may be under-represented, and there may be medications where use is on the decline (e.g., codeine). Our definition of exposure required only a single mention of a drug name with a dose, route or frequency, which may have false positives, and our EHR data may not be complete, particularly as many patients receive some of their care outside this institution. Also, while EHR data are robust for determining medication start dates, discontinuation dates are difficult to discern, and thus we have not calculated length of exposure and cannot comment on whether pediatric patients were treated briefly or chronically with these drugs. Despite these limitations, the data likely represent a reasonable approximation for the current state of the field of pharmacogenomics and pediatric exposures to drugs with actionable drug–CYP interactions.

Based on our analysis, it is apparent that there is much work to do in the field of pediatric pharmacogenomics. For most drugs with known interactions with CYP enzymes, data must be collected in a robust fashion to determine the veracity of those associations in children. There are a few drugs with robust evidence, namely codeine and PPIs. The former is now contraindicated in children under 12 and is likely to be used less and less in pediatric patients. PPIs are very frequently used, and efforts can move forward towards implementation of *CYP2C19*-guided therapy. It may be appropriate to extrapolate from adult data to non-neonatal pediatric patients for the ondansetron–*CYP2D6* interaction. For the remaining drug–CYP interactions, further work must be completed prior to implementation. Given the fact that many drugs with known interactions are commonly used in children, these high-use drugs provide a reasonable starting point.

## 4. Materials and Methods 

### 4.1. Identification of CYP-Associated Drugs with High Levels of Evidence

In order to generate a list of candidate drugs to assess in the pediatric population, all gene–drug pairs from the CPIC website were downloaded on 14 August 2017 [12,15]. The downloaded table included CPIC and PharmGKB level of evidence for each gene–drug pair. The complete list of all gene–drugs pairs was filtered to include only those with CYP in the gene name. The list was then further filtered to include only those with PharmGKB level 1A, 1B, 2A or 2B evidence and CPIC level A, A/B or B levels. 

### 4.2. Determining the Pediatric Exposures to CYP-Associated Drugs

To determine the number of unique pediatric patients exposed to the actionable CYP-associated drugs identified from the CPIC table, we used the Synthetic Derivative (SD), the de-identified electronic health records database at Vanderbilt University Medical Center. The study was reviewed by the Vanderbilt Institutional Review board and determined to be non-human subjects research. Exposure to the medication of interest was defined as one or more mention of the drug name, in conjunction with a dose, route, strength or frequency, using MedEx [118]. For this study, we restricted mentions to those occurring in the 10-year period from 2006 to 2016. These date ranges are approximate given that dates in the SD are de-identified, and every entry in each individual’s record is date shifted with a random (but consistent) number of days up to one year backwards. This date shifting should not meaningfully impact our convenience sample. Individuals were included as pediatric exposures if their age was <18 years on the date of the first exposure to the medication of interest.

To further characterize the pediatric exposures, we collected additional demographic data for each cohort of patients exposed to a drug of interest. The age of each individual on the date of first drug mention was collected and is reported as the median and interquartile range, as well as the distribution of sex, race and ethnicity for those exposed. Race and ethnicity are coded using administratively defined variables in the electronic health record. 

### 4.3. Literature Review for Drug–CYP Interactions in Children

For the CYP-associated drugs with more than 5000 pediatric patients exposed over a 10-year period in our electronic health records cohort, we performed a focused literature review of the evidence for the drug–CYP association in pediatric patients. Reports were included if they were written in English (or an English language translation was available), if some or the entire study cohort was <21 years of age, and if one or more CYP enzyme was evaluated through genotyping or phenotyping. Pharmacokinetic studies that measured drug and metabolite concentrations, pharmacodynamic studies that measured drug response, and clinical implementation studies that used pharmacogenomic data to guide prescribing were included. Findings are described using the standard nomenclature suggested by CPIC [119].

## Figures and Tables

**Table 1:** Number of Pediatric Patients Exposed to each CYP-Associated Drug in a 10-year Period.

Rank	Drug	*N* Children Exposed	Median (IQR) Age at First Exposure (Years)	*n* (%) Male	*n* (%) White	*n* (%) Black/African American	*n* (%) Asian	*n* (%) Other	*n* (%) Unknown Race	*n* (%) Hispanic or Latino	*n* (%) Not Hispanic or Latino	*n* (%) Unknown Ethnicity
**1**	ondansetron	114,059	6 (2–13)	61,339 (53.8%)	83,687 (73.4%)	21,232 (18.6%)	2188 (1.9%)	1321 (1.2%)	5631 (4.9%)	11,161 (9.8%)	98,732 (86.6%)	4166 (3.7%)
**2**	oxycodone	30,701	11 (4–16)	16,330 (53.2%)	22,696 (73.9%)	4977 (16.2%)	623 (2.0%)	203 (0.7%)	2202 (7.2%)	2309 (7.5%)	26,506 (86.3%)	1886 (6.1%)
**3**	codeine	21,086	4 (1–8)	11,445 (54.3%)	15,482 (73.4%)	3928 (18.6%)	445 (2.1%)	254 (1.2%)	977 (4.6%)	1986 (9.4%)	18,387 (87.2%)	713 (3.4%)
**4**	omeprazole	21,056	10 (4–15)	10,355 (49.2%)	15,531 (73.8%)	2479 (11.8%)	320 (1.5%)	91 (0.4%)	2635 (12.5%)	1344 (6.4%)	17,356 (82.4%)	2356 (11.2%)
**5**	lansoprazole	17,451	5 (0–11)	9261 (53.1%)	13,345 (76.5%)	1663 (9.5%)	217 (1.2%)	132 (0.8%)	2094 (12.0%)	680 (3.9%)	14,797 (84.8%)	1974 (11.3%)
**6**	sertraline	10,417	14 (10–16)	4627 (44.4%)	7931 (76.1%)	1075 (10.3%)	116 (1.1%)	33 (0.3%)	1262 (12.1%)	379 (3.6%)	8919 (85.6%)	1119 (10.7%)
**7**	amitriptyline	7918	13 (10–16)	3020 (38.1%)	5658 (71.5%)	867 (10.9%)	65 (0.8%)	35 (0.4%)	1293 (16.3%)	340 (4.3%)	6431 (81.2%)	1147 (14.5%)
**8**	citalopram	7528	13 (8–16)	3751 (49.8%)	5762 (76.5%)	847 (11.3%)	90 (1.2%)	36 (0.5%)	793 (10.5%)	359 (4.8%)	6489 (86.2%)	680 (9.0%)
**9**	risperidone	5485	11 (7–15)	3793 (69.2%)	3890 (70.9%)	927 (16.9%)	59 (1.1%)	23 (0.4%)	586 (10.7%)	182 (3.3%)	4767 (86.9%)	536 (9.8%)
**10**	escitalopram	5087	15 (12–17)	2049 (40.3%)	3990 (78.4%)	396 (7.8%)	73 (1.4%)	22 (0.4%)	606 (11.9%)	181 (3.6%)	4364 (85.8%)	542 (10.7%)
**11**	atomoxetine	3681	11 (8–14)	2581 (70.1%)	2776 (75.4%)	350 (9.5%)	29 (0.8%)	16 (0.4%)	510 (13.9%)	86 (2.3%)	3124 (84.9%)	471 (12.8%)
**12**	paroxetine	3445	7 (2–15)	1641 (47.6%)	2384 (69.2%)	532 (15.4%)	98 (2.8%)	15 (0.4%)	416 (12.1%)	376 (10.9%)	2786 (80.9%)	283 (8.2%)
**13**	tramadol	2731	14 (5–17)	1228 (45.0%)	2051 (75.1%)	361 (13.2%)	44 (1.6%)	14 (0.5%)	261 (9.6%)	142 (5.2%)	2366 (86.6%)	223 (8.2%)
**14**	methadone	2559	0 (0–4)	1447 (56.5%)	1901 (74.3%)	376 (14.7%)	32 (1.3%)	22 (0.9%)	228 (8.9%)	184 (7.2%)	2159 (84.4%)	216 (8.4%)
**15**	tacrolimus	2253	8 (3–13)	1132 (50.2%)	1434 (63.6%)	440 (19.5%)	67 (3.0%)	15 (0.7%)	297 (13.2%)	170 (7.5%)	1854 (82.3%)	229 (10.2%)
**16**	nortriptyline	2179	12 (8–16)	864 (39.7%)	1527 (70.1%)	246 (11.3%)	33 (1.5%)	14 (0.6%)	359 (16.5%)	111 (5.1%)	1754 (80.5%)	314 (14.4%)
**17**	warfarin	2091	9 (3–15)	1107 (52.9%)	1496 (71.5%)	314 (15.0%)	50 (2.4%)	15 (0.7%)	216 (10.3%)	150 (7.2%)	1804 (86.3%)	137 (6.6%)
**18**	phenytoin	1725	12 (3–16)	980 (56.8%)	1341 (77.7%)	255 (14.8%)	31 (1.8%)	15 (0.9%)	83 (4.8%)	99 (5.7%)	1542 (89.4%)	84 (4.9%)
**19**	mirtazapine	1578	12 (8–16)	943 (59.8%)	1155 (73.2%)	211 (13.4%)	26 (1.6%)	4 (0.3%)	182 (11.5%)	72 (4.6%)	1352 (85.7%)	154 (9.8%)
**20**	venlafaxine	1407	15 (8–17)	558 (39.7%)	1097 (78.0%)	127 (9.0%)	31 (2.2%)	7 (0.5%)	145 (10.3%)	62 (4.4%)	1232 (87.6%)	113 (8.0%)
**21**	clopidogrel	1131	7 (2–14)	605 (53.5%)	778 (68.8%)	185 (16.4%)	35 (3.1%)	5 (0.4%)	128 (11.3%)	92 (8.1%)	957 (84.6%)	82 (7.3%)
**22**	imipramine	877	11 (8–13)	512 (58.4%)	533 (60.8%)	112 (12.8%)	6 (0.7%)	5 (0.6%)	221 (25.2%)	26 (3.0%)	643 (73.3%)	208 (23.7%)
**23**	celecoxib	795	14 (6–16)	351 (44.2%)	605 (76.1%)	85 (10.7%)	14 (1.8%)	1 (0.1%)	90 (11.3%)	45 (5.7%)	678 (85.3%)	72 (9.1%)
**24**	dexlansoprazole	554	14 (9–16)	264 (47.7%)	444 (80.1%)	49 (8.8%)	13 (2.3%)	2 (0.4%)	46 (8.3%)	35 (6.3%)	480 (86.6%)	39 (7.0%)
**25**	doxepin	436	13 (6–17)	194 (44.5%)	294 (67.4%)	69 (15.8%)	8 (1.8%)	1 (0.2%)	64 (14.7%)	19 (4.4%)	356 (81.7%)	61 (14.0%)
**26**	fluvoxamine	414	13 (10–16)	237 (57.2%)	324 (78.3%)	34 (8.2%)	7 (1.7%)	1 (0.2%)	48 (11.6%)	7 (1.7%)	363 (87.7%)	44 (10.6%)
**27**	voriconazole	323	9 (3–15)	183 (56.7%)	247 (76.5%)	44 (13.6%)	8 (2.5%)	1 (0.3%)	23 (7.1%)	25 (7.7%)	280 (86.7%)	18 (5.6%)
**28**	rabeprazole	213	14 (8–16)	96 (45.1%)	161 (75.6%)	22 (10.3%)	1 (0.5%)	3 (1.4%)	26 (12.2%)	10 (4.7%)	180 (84.5%)	23 (10.8%)
**29**	clomipramine	188	13 (9–15)	115 (61.2%)	150 (79.8%)	14 (7.4%)	4 (2.1%)	0 (0.0%)	20 (10.6%)	10 (5.3%)	162 (86.2%)	16 (8.5%)
**30**	tamoxifen	83	11 (4–15)	48 (57.8%)	59 (71.1%)	9 (10.8%)	1 (1.2%)	0 (0.0%)	14 (16.9%)	4 (4.8%)	67 (80.7%)	12 (14.5%)
**31**	nevirapine	81	6 (0–12)	40 (49.4%)	27 (33.3%)	40 (49.4%)	0 (0.0%)	1 (1.2%)	13 (16.0%)	4 (4.9%)	66 (81.5%)	11 (13.6%)
**32**	efavirenz	56	13 (5–16)	34 (60.7%)	27 (48.2%)	24 (42.9%)	1 (1.8%)	0 (0.0%)	4 (7.1%)	3 (5.4%)	50 (89.3%)	3 (5.4%)
**33**	quinidine	56	10 (3–14)	35 (62.5%)	45 (80.4%)	4 (7.1%)	1 (1.8%)	1 (1.8%)	5 (8.9%)	3 (5.4%)	50 (89.3%)	3 (5.4%)
**34**	desipramine	49	13 (3–17)	21 (42.9%)	37 (75.5%)	8 (16.3%)	1 (2.0%)	0 (0.0%)	3 (6.1%)	2 (4.1%)	44 (89.8%)	3 (6.1%)
**35**	trimipramine	18	8 (2–13)	11 (61.1%)	13 (72.2%)	2 (11.1%)	0 (0.0%)	0 (0.0%)	3 (16.7%)	0 (0.0%)	15 (83.3%)	3 (16.7%)
**36**	brexpiprazole	13	16 (13–17)	4 (30.8%)	9 (69.2%)	1 (7.7%)	0 (0.0%)	0 (0.0%)	3 (23.1%)	0 (0.0%)	10 (76.9%)	3 (23.1%)
**37**	tropisetron	12	3 (1–10)	7 (58.3%)	10 (83.3%)	1 (8.3%)	0 (0.0%)	0 (0.0%)	1 (8.3%)	0 (0.0%)	11 (91.7%)	1 (8.3%)
**38**	eliglustat	6	11 (8–15)	3 (50.0%)	6 (100.0%)	0 (0.0%)	0 (0.0%)	0 (0.0%)	0 (0.0%)	0 (0.0%)	6 (100.0%)	0 (0.0%)
**39**	protriptyline	6	17 (14–17)	1 (16.7%)	4 (66.7%)	0 (0.0%)	0 (0.0%)	0 (0.0%)	2 (33.3%)	0 (0.0%)	4 (66.7%)	2 (33.3%)
**40**	acenocoumarol	3	13 (12–15)	1 (33.3%)	2 (66.7%)	0 (0.0%)	0 (0.0%)	0 (0.0%)	1 (33.3%)	0 (0.0%)	2 (66.7%)	1 (33.3%)
**41**	phenprocoumon	3	13 (12–15)	1 (33.3%)	2 (66.7%)	0 (0.0%)	0 (0.0%)	0 (0.0%)	1 (33.3%)	0 (0.0%)	2 (66.7%)	1 (33.3%)

*n*—Number; IQR—Interquartile Range.

**Table 2:** Evidence for CYP–drug Interactions in Pediatrics.

Drug	Gene	Variant(s) Assayed	Population	*n*	Significant Result	Results	Ref.
Oxycodone	*CYP2D6*	*2–*11, *14, *15, *17–*20, *35, *40–*42, *44, duplication	2–17-year-olds undergoing painful orthopedic, thoracic, urology and colorectal procedures	30	Yes	After oxycodone exposure, CYP2D6 normal metabolizers had greater oxymorphone exposure than poor or intermediate metabolizers	[18]
Codeine	*CYP2D6*	CYP2D6 phenotype	15–74-year-old healthy volunteers	132	Yes	After codeine administration, CYP2D6 poor metabolizers had lower formation of morphine versus normal metabolizers	[19]
Codeine	*CYP2D6*	*2–*5, *9, *10, *17	3–12-year-olds undergoing adenotonsillectomy	48	Yes	After codeine administration, CYP2D6 poor metabolizers had reduced formation of morphine	[20]
Codeine	*CYP2D6*	not stated	case report: breastfed neonate	1	--	Fatal opioid poisoning in a breastfed neonate whose codeine-prescribed mother was a CYP2D6 ultra-rapid metabolizer	[21]
Codeine	*CYP2D6*	not stated	case report: post-tonsillectomy codeine with apnea and brain injury	1	--	2-year-old child with codeine toxicity after tonsillectomy was CYP2D6*1/*2	[22]
Codeine	*CYP2D6*	not stated	mothers and infants with codeine exposure	72	Yes	Two of 17 mothers whose infants exhibited severe neonatal toxicity were CYP2D6 ultra-rapid metabolizers in combination with UGT2B7*2/*2	[23]
Codeine	*CYP2D6*	not stated	case report: fatality in child with adenotonsillectomy	1	--	Death in a 2-year-old boy prescribed codeine for analgesia after adenotonsillectomy and with CYP2D6 ultra-rapid metabolizer phenotype	[24]
Codeine	*CYP2D6*	*3–*6	case report: fatality and respiratory failure in 3-year-old monozygotic twin brothers	2	--	Death of one twin and respiratory failure with successful resuscitation of the other twin after administration of slow-release codeine cough medicine in CYP2D6 normal metabolizers	[25]
Codeine	*CYP2D6*	not stated	case report: fatal or life-threatening codeine exposures after tosillectomy	3	--	Two fatalities and one case of respiratory failure after post-tonsillectomy codeine exposure; one decedent was a CYP2D6 ultra-rapid metabolizer and the resuscitated child was a CYP2D6 normal metabolizer	[26]
Codeine	*CYP2D6*	*2–*10, *12, *14 *17, *29, *41, duplication	1–17-year-olds with obstructive sleep apnea syndrome who underwent adenotonsillectomy	21	No	*CYP2D6* genotype did not predict change in the rate of desaturation and in the nadir oxygen saturation values	[27]
Codeine	*CYP2D6*	*2–*10,*12, *14, *17, *29, *41, duplication	breastfeeding mothers using codeine and their infants	111	Yes	Maternal risk genotypes in *CYP2D6* and *ABCB1* were significantly associated with the adverse outcomes in infants	[28]
Codeine	*CYP2D6*	not stated	case report: codeine related fatality	3	--	One of the three cases of codeine fatality was a CYP2D6 normal metabolizer	[29]
Codeine	*CYP2D6*	*2–*11, *14, *15, *17–*20, *35, *40–*42, *44, duplication	6–15-year-olds undergoing tonsillectomy	134	Yes	Increased adverse drug reaction risk was associated with the presence of one or more full function *CYP2D6* alleles	[30]
Codeine	*CYP2D6*	Affymetrix DMET Plus GeneChip microarray, duplication	Patients with sickle cell disease	830	--	None of the patients with an ultra-rapid or poor metabolizer *CYP2D6* genotype were prescribed codeine	[31]
Omeprazole	*CYP2C19*	*2–*8, *10, *12, *17	2–16-year-olds with therapeutic need for acid-modifying therapy	23	No	No relationship between *CYP2C19* genotype and pharmacokinetic parameters (area under curve or clearance)	[32]
Lansoprazole	*CYP2C19*	*2, *3, *8, *9, *17	6–17-year-olds with poor asthma control while treated with inhaled corticosteroids	279	Yes	Upper respiratory tract infections and strep throat were more frequent in CYP2C19 poor metabolizers than normal metabolizers or placebo	[33]
Lansoprazole	*CYP2C19*	*1, *2, *3	0–18-year-olds with *H. pylori* infection	100	No	No significant difference in cure rates in CYP2C19 normal vs. poor metabolizers	[34]
Lansoprazole	*CYP2C19*	*2, *3, *8–*10, *17	6–17-year-olds with poor asthma control while treated with inhaled corticosteroids	279	Yes	CYP2C19 poor metabolizers exposed to lansoprazole had worsening of asthma control	[35]
PPI	*CYP2C19*	*2, *8, *17	Children with gastroesophageal reflux refractory to PPI therapy	74	Yes	Increased acid exposure (lower intra-gastric pH) in CYP2C19 ultra-rapid metabolizers than non-ultra-rapid metabolizers	[36]
Amitriptyline	*CYP2C19*	not stated	case report: 6-year-old child with amitriptyline overdose	1	--	Patient survived a chronic 10-fold amitryptine overdose; genotyping revealed CYP2C19*1/*1	[37]
Amitriptyline	*CYP2D6*	not stated	case report: 6-year-old child with amitriptyline overdose	1	--	Patient survived a chronic 10-fold amitryptine overdose; genotyping revealed CYP2D6*1/*41	[37]
Citalopram	*CYP2C19*	*2, *3	15–20-year-olds treated with citalopram for major depressive disorder or dysthymia	19	No	No difference in citalopram pharmacokinetics by *CYP2C19* genotype	[38]
Citalopram	*CYP2D6*	*2–*6, duplicaton	15–20-year-olds treated with citalopram for major depressive disorder or dysthymia	19	No	No difference in citalopram pharmacokinetics by *CYP2D6* genotype	[38]
Citalopram & Escitalopram	*CYP2C19*	*2–*5	15–84-year-olds with citalopram or escitalopram therapeutic drug monitoring	83	Yes	CYP2C19 intermediate metabolizers had impaired metabolism of citalopram and *S*-citalopram compared to normal metabolizers	[39]
Escitalopram	*CYP2C19*	*2,*3,*17	4–45-year-olds with ASD	89	No	No significant difference in citalopram dose by CYP2C19 metabolizer status	[40]
Risperidone	*CYP2D6*	*3–*7, duplication	5–17-year-olds with pervasive developmental disorder	25	Yes	Serum prolactin level was positively correlated with CYP2D6 function	[41]
Risperidone	*CYP2D6*	*3–*5, duplication	4–15-year-olds treated with risperidone for psychiatric or neurodevelopmental conditions	19	No	In pharmacokinetic analysis, one outlier identified was found to be a CYP2D6 poor metabolizer	[42]
Risperidone	*CYP2D6*	*3–*6, duplication	3–21-year-olds with ASD	45	Yes	*CYP2D6* polymorphisms were associated with risperidone-induced increase in body mass index or waist circumference	[43]
Risperidone	*CYP2D6*	*2–*11, *14, *15, *17–*20, *40–*42, duplication	3–18-year-olds treated with risperidone for a neuropsychiatric disorder	28	No	Clearance estimates for a 1-compartment mixture model were highest for CYP2D6 normal metabolizers and lowest for poor metabolizers	[44]
Risperidone	*CYP2D6*	*3, *4, *5, *6, duplication	10–19-year-old males with ASD or disruptive behavior disorders	47	No	No statistically signifant difference in prolactin level by CYP2D6 functional status	[45]
Risperidone	*CYP2D6*	*3, *4, *5, *6, *9, *10, *41	8–89-year-olds with risperidone TDM	190	Yes	Higher risperidone serum concentration in those with reduced CYP2D6 function	[46]
Risperidone	*CYP2D6*	*2–*11, *14, *15, *17–*20, *25, *26, *29, *30, *31, *35–*37, *40, *41, *43, *52, duplication	3–18-year-olds with ASD or pervasive developmental disorders	40	Yes	Higher risperidone plasma concentrations and risperidone:9-hydroxyrisperidone ratio in CYP2D6 poor metabolizers, but no significant association between the CYP2D6 function and clinical response or adverse effects	[47]
Risperidone	*CYP2D6*	*4	9–20-year-olds with schizophrenia or bipolar disorder	81	Yes	Significantly higher weight gain in those with CYP2D6*4	[48]
Risperidone	*CYP2D6*	*10	8–20-year-olds treated with risperidone for mental or behavioral disorder	120	No	No significant association between plasma prolactin levels and CYP2D6*10 allele	[49]
Risperidone	*CYP2D6*	*4, *5, *10, *41	3–19-year-olds with ASD	147	No	No significant correlation of prolactin levels and *CYP2D6* genotype	[50]
Risperidone	*CYP2D6*	*2–*11, *15, *29, *33, *41, duplication	3–20-year-olds with ASD	84	Yes	Higher risperidone plasma concentration risperidone: 9-hydroxyrisperidone ratio among those with reduced CYP2D6 function	[51]
Risperidone	*CYP2D6*	*10	8–20-year-olds treated with risperidone for mental and behavioral disorders	120	Yes	Obese/overweight and hypertension were associated with CYP2D6*10	[52]
Risperidone	*CYP2D6*	*3–*6, *9, *10, *41, duplication	9–93-year-olds with risperidone TDM	425	Yes	Risperidone: 9-hydroxyrisperidone concentration ratio correlated with CYP2D6 function	[53]
Risperidone	*CYP2D6*	Affymetrix DMET Plus GeneChip microarray	Children with ASD (median age 8.8 (IQR 3.4–18.6) years)	102	Yes	*CYP2D6* variants were associated with risperidone plasma concentration and the risperidone: 9-hydroxyrisperidone ratio	[54]
Risperidone	*CYP2D6*	*4, *5, *10, *41	Children with ASD (median age 10 (IQR 7–12.15) years)	97	Yes	Plasma levels of risperidone were significantly higher in individuals with decreased CYP2D6 function	[55]

*n*—Number included in pharmacogenomic study; Ref.—reference; PPI—Proton pump inhibitor, including omeprazole, lansoprazole, esomeprazole, and pantoprazole; ASD—Autism Spectrum Disorder; TDM—Therapeutic Drug Monitoring.
